# Autoimmune thyroid disease

**DOI:** 10.1007/s12020-020-02188-6

**Published:** 2020-01-18

**Authors:** Anthony Weetman

**Affiliations:** Dept of Human Metabolism, University of Sheffield, Sheffield, UK

I have been asked to give a brief summary of my perspective on the initial development of research in autoimmune thyroid disease and Marian Ludgate’s contribution to that, as well as outlining thoughts on future progress in the field. This is therefore a personal article which commemorates our time together in Cardiff in the 1980s that has led to a very enjoyable and fruitful collaboration over three decades. A detailed review is available elsewhere of the early period of thyroid research [1] and only minimal references are contained in this article.

The discovery of the phenomenon of autoimmunity itself depended on the thyroid. The prevailing dogma of ‘horror autotoxicus’, first properly defined by Paul Ehrlich, was overturned in 1956 by seminal work from Ernest Witebsky and Noel Rose who showed that rabbits immunised with thyroid extract and adjuvant developed thyroiditis and thyroid autoantibodies [2]. This was seized on by Ivan Roitt and Deborah Doniach who had been studying Hashimoto’s thyroiditis and who first described such autoantibodies in the serum of these patients [3]. Hashimoto’s thyroiditis was deemed such a rare disease at the time that it took Rose a further year to collect sufficient patient samples to repeat this finding.

Description of the two basic thyroid autoantigens, thyroglobulin and a microsomal protein (identified as thyroid peroxidase much later), soon followed. Coupled with the development of radioimmunoassays and the purification of hypothalamic releasing hormones, this led to rapid developments in thyroid autoimmunity in many laboratories over the next two decades, including that of Reg Hall in Newcastle who highlighted an important genetic contribution with the finding of a high frequency of thyroid autoantibodies in the families of disease-affected probands [4]. Rose refined his animal model to work in rodents and, alongside other groups such as that of William Weigle, showed an aetiological contribution from both major histocompatibility complex (MHC) and non-MHC genes as well as from environmental factors, and dissected out the contribution of T and B cells, providing the first direct evidence of T cell-mediated cytotoxicity as an underlying pathogenetic feature [5]. Also during this time, the laboratories of Max McKenzie and Joe Kriss provided the evidence that the serum ‘long-acting thyroid stimulator’ in Graves’ disease, described in 1956 by Purves and Adams, was a TSH-receptor autoantibody.

The major question at the time, and one which still remains to be fully addressed, was why only certain individuals with the appropriate genetic and environmental background go on to develop autoimmune thyroid disease, a conundrum underscored by the discovery of a high prevalence of subclinical disease that did not appear to progress. Another seminal finding from Rose, namely that genetically predisposed normal mice nonetheless had thyroid-reactive T cell [6], indicated that there must be some sort of ongoing protective mechanism to prevent loss of self-tolerance.

Much of thyroid autoimmunity research at this time depended heavily on developments in basic murine immunology, and the prevailing theory to explain tolerance to self-antigens in the 1970s was developed by Richard Gershon, who proposed the existence of a suppressor class of T cells. Thyroidologists were quick to seize upon this idea, which was bolstered by John Penhale’s discovery of a class of T cells with apparently such properties which could be transferred from healthy rats to neonatal rats subjected to thymectomy and sublethal irradiation, preventing the development of autoimmune thyroiditis (such surgery and irradiation at a critical stage in development removed these cells, permitting disease to occur) [7].

The major limitation to research then, as now, was that the frequency of thyroid-reactive T cells in the accessible circulation is so low that methods to study autoreactive responses were difficult to develop. In part this is because the lymphocytic infiltrate in the thyroid itself is the main focus for the autoimmune response, and also because over time it is to be expected that autoreactivity will decline as the source of autoantigen is destroyed. Despite these limitations, the laboratory of Robert Volpé produced evidence for thyroid antigen-specific T suppressor cells in a series of experiments based around tests for migration inhibition factor (MIF). This lymphokine was proposed to be released by autoreactive T cells on contact with autoantigen and could be measured by calculating the migration of T lymphocytes packed into a capillary tube. Healthy donor T cells were able to prevent MIF production by lymphocytes from individuals with thyroiditis in an antigen-specific manner and were therefore identified as thyroid-specific T suppressor cells [8]. The belief that a defect in these suppressor cells underlay all autoimmune endocrinopathies became the established view by the early 1980s.

At the same time, a major breakthrough in the difficulty of studying T cells appeared in the availability of monoclonal antibodies that could identify human T cell subsets; such antibodies had already proved invaluable in murine immunology. Marian and I were by this time new recruits to the Hall lab, which had moved to Cardiff. Working with Alan McGregor, Marian was the first to show that in Graves’ disease there was a reduced level of CD8+ (loosely identified as ‘suppressor/cytotoxic’) T cells returning to normal levels after carbimazole treatment and high levels of HLA-DR+ ‘activated’ T cells returning to normal after treatment [9], although it is now clear that such broad phenotypic markers can only hint at what may really be going on functionally. As well as demonstrating disordered T-cell regulation in Graves’ disease, this study showed that antithyroid drugs had immunomodulatory properties.

These findings clearly demanded further dissection: exactly what role did the altered T cells play? So Marian’s attention turned to the MIF assay, which despite her usual meticulous rigour proved to be impossible to replicate. It became obvious that the suppressor cell assay as described used non-physiological conditions; as well as being an allogeneic system, tests were conducted on a bench at room temperature rather than in an incubator at 37 °C [10]. This negative finding really marked the beginning of the end of the theory of an antigen-specific T suppressor cell defect in autoimmune thyroid disease. Failure to identify a proposed I-J region on the MHC crucial to the genetic control of murine T suppressor cells proved the final blow to the whole concept of these cells, which then fell from favour.

Yet the results of the Penhale model demanded explanation and this was pursued relentlessly by Shimon Sakaguchi’s lab who used variants of the model to define, in 1995, a CD4+, CD25+ regulatory subset of T cells that were critical to the prevention of a variety of autoimmune diseases in mice. Subsequent work from his lab and from Ethan Shevach, Don Mason, and Fiona Powrie further characterised these T regulatory cells as FoxP3+ and identified such cells in man; these cells differ in several aspects to the originally conceived T suppressor cells. We now know that several types of T regulatory cells exist and their therapeutic potential is beginning to be realised.

Marian left Cardiff in the mid-1980s and joined the lab of Gilbert Vassart where the molecular identification of thyroid antigens was the major focus for the next decade, as it was for other leading labs such as that of Basil Rapoport. The genetics of thyroid autoimmunity was also subjected to a molecular approach and has proved far more complex than originally imagined. New insights especially from the study of cytokines has allowed both an appreciation of the role thyroid cells themselves play in the autoimmune process and the description of peripheral tolerance as a second regulatory mechanism for self-tolerance, operating through thyrocyte HLA-DR expression in the absence of a costimulatory signal.

What of the future? We are on the cusp of using our accumulated knowledge to develop a precision medicine approach to the classification of autoimmune thyroid disease. It has already been shown that there is an important but hitherto unsuspected subclass of Hashimoto’s thyroiditis based on IgG4 levels. Proteomic technology will allow measurement of a number of autoantibodies (multiplexing) based on addressable microbeads or nanobarcoded particles. The phenomenon of ‘autoimmune escalation’ may be used to identify which antibody clusters better mark temporary and permanent damage, especially when combined with genetic markers. Several other developments I would hope for include (1) identification of B cell-surface autoantibodies and the autoantigenic peptides presented to T cells by these overlooked antigen-presenting cells; (2) definitive immunotherapy for Graves’ disease targeted at the thyroid cell’s role in the autoimmune process or at T regulatory cells; (3) renewed focus on the target organ and exactly how thyroid cells are destroyed or defend themselves from destruction and (4) a better understanding of the contribution of the microbiome to aetiology. And of course that enduring enigma, thyroid ophthalmopathy, which has been Marian’s most recent interest, will be fully understood, but that is the subject a different article!
